# Technical Note: Development of 3D‐printed breast phantoms for end‐to‐end testing of whole breast volumetric arc radiotherapy

**DOI:** 10.1002/acm2.12976

**Published:** 2020-08-15

**Authors:** Laurence Delombaerde, Saskia Petillion, Caroline Weltens, Robin De Roover, Truus Reynders, Tom Depuydt

**Affiliations:** ^1^ Department of Oncology KU Leuven Leuven Belgium; ^2^ Department of Radiation Oncology University Hospitals Leuven Leuven Belgium

**Keywords:** 3D‐printing, anthropomorphic phantom, breast VMAT‐SIB, commissioning, dose verification

## Abstract

End‐to‐end testing of a new breast radiotherapy technique preferably requires realistic phantom geometries, which is challenging to achieve using currently commercially available solutions. We have developed a series of three‐dimensional (3D)‐printed breast phantoms, with ionization chamber and radiochromic film inserts, which can be attached to a commercial anthropomorphic thorax phantom. A contoured left breast from a patient’s planning CT was mapped onto a CT of the CIRS E2E thorax phantom (CIRS Inc.) and cropped to fit the surface. Four versions of the breast were 3D printed, containing a cavity for an ionization chamber and slits for radiochromic film insertion in the three cardinal planes, respectively. The phantoms were fully compatible with surface scanning technology used for setup. The phantoms were validated using a whole‐breast volumetric modulated arc therapy protocol with a simultaneous integrated boost to the tumor bed (VMAT‐SIB). Six patient plans and one original plan on the breast phantom were verified with planar portal imaging, point dose, and film measurements in the MultiCube phantom and planar γ‐analysis using ArcCHECK diode array. Six patient plans were recalculated on the breast phantom (hybrid plans) and delivered with point dose and film measurements with 3% (local)/2 mm γ‐analysis. One complete end‐to‐end test on the breast phantom was performed. All plan quality verifications had point dose differences below 2.4% from the calculated dose and γ‐agreement scores (γAS) > 87.3% for film measurements in the MultiCube, portal dosimetry, and ArcCHECK. Point dose differences in the 3D‐printed phantoms were below 2.6% (median −1.4%, range −2.6%; 0.3%). Median γAS was 96.4% (range 80.1%–99.7%) for all film inserts. The proposed 3D‐printed attachable breast dosimetry phantoms have been shown to be a valuable tool for end‐to‐end testing of a new radiotherapy protocol. The workflow described in this report can aid users to create their own phantom‐specific breast 3D‐printed phantoms.

## 1 INTRODUCTION

The validation of new volumetric arc radiotherapy (VMAT) or intensity‐modulated radiotherapy (IMRT) protocols is generally performed via end‐to‐end tests, where the entire radiotherapy workflow from planning to delivery is verified. TG‐119 recommends the use of *target and structure geometries along with the target doses and dose constraints that are likely to be encountered in the clinic* for IMRT commissioning.^1^ However, most dosimetry phantoms lack the specific anatomy required for a “realistic” end‐to‐end test, namely, all steps from patient imaging, to contouring, to setup using surface scanning technology, for breast cancer plans.

Existing anthropomorphic phantoms with breast attachments are the Alderson Radiation Therapy phantom (Radiology Support Devices Inc., Carson, CA, US) and its earlier version, the Alderson RANDO phantom. However, they only contain cylindrical extrusions for thermoluminescent dosimeters (TLDs), not widely available in radiotherapy departments. Others, such as the end‐to‐end (E2E) SBRT Thorax phantom (CIRS Inc., Norfolk, VA, US), have multiple holes for ionization chamber (IC) and dosimetric film insertion, yet lack external features mimicking breasts. External features are also lacking in the thorax phantom of the Imaging and Radiation Oncology Core group (IROC) distributed for quality assurance (QA).^2^ Moreover, all these phantoms have a shiny coating, cylindrical symmetry, or both, limiting the use of surface scanning technology used for patient setup,^3^, ^4^ which is increasingly being used for patient setup,^5^ and whose positioning accuracy should be incorporated into commissioning.

Recent dispersion of low‐cost three‐dimensional (3D)‐printing technology has allowed for the development of (patient) specific phantoms in radiotherapy.^6^, ^7^, ^8^ This work describes the development of a series of end‐to‐end left breast attachments to the CIRS SBRT thorax phantom, using a mid‐range 3D printer, allowing for commissioning of breast radiotherapy treatment techniques. All phantoms are fully compatible with surface scanning technology for accurate positioning and have either a film or IC insert centrally located in the breast. The phantoms were validated by performing hybrid plan measurements — where patient plans are recalculated on the phantom — for six VMAT breast treatments with simultaneous integrated boost (VMAT‐SIB). Additionally, an end‐to‐end test was performed whereby a VMAT‐SIB plan was created on a simulation scan of the phantoms, the phantoms were positioned with surface scanning technology and the plan was delivered on the breast phantoms with film and IC insert.

## 2 MATERIALS AND METHODS

### 2.A Development of 3D‐printed phantoms

**Fig. 1:**
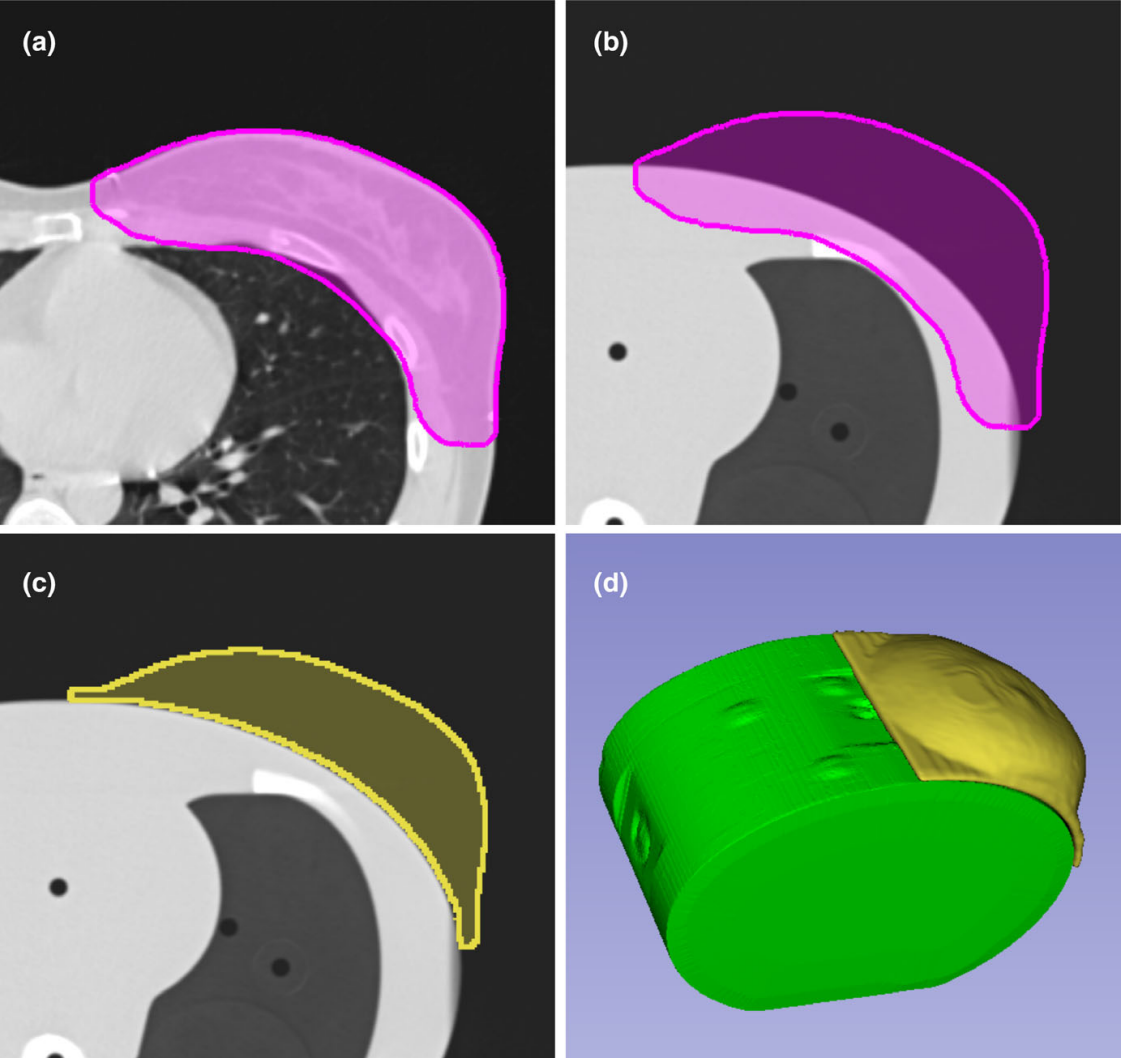
Model generation workflow. The contoured breast PTV (a) was mapped onto a planning CT of the CIRS SBRT thorax E2E phantom (b). Overlap with the thorax phantom was cropped (c). The final model shown in the three‐dimensional view and its location on the thorax phantom where it uniquely fits in the phantom’s lateral indentation (d).

To obtain a realistic breast shape, the contoured breast volume on the computed tomography (CT) of a patient was imported into 3DSlicer (version 4.10).^9^ The breast volume was mapped onto a planning CT of the CIRS E2E SBRT thorax phantom, as shown in Fig. 1. The breast volume was then cropped to create a tight fit with the CIRS phantom. The phantom base shape of 698 cc was then exported as a stereolithography file (.stl). To create the final phantoms, 0.3‐mm‐wide slits were cut out using FreeCAD^10^ (version 0.17) in the three cardinal planes (ie, sagittal, coronal, and axial). The fourth phantom was created by hollowing out a cylindrical cavity for the IC phantom (⌀ 6.75 mm).

The phantoms were printed in red polylactic acid (PLA) (ICE Filaments, Belgium) on a Raise3D N2 Plus 3D printer (Raise3D, The Netherlands) with 80% infill and two outlines. In a preparatory step cubes (5 × 5 × 5 cm^3^) with varying infill were printed and scanned at 120 kVp, slice thickness 1 mm on a Somatom Sensation Open (Siemens Healthcare GmbH, Erlangen, Germany), and the average Hounsfield Unit determined in a 20 cm^3^ region of interest. From these the infill percentage was chosen as a compromise between water equivalence (80% resulted in an average of −160 HU, SD 6 HU) and possible warping of the phantoms, known to increase for higher infill percentages.^11^ To avoid support structures in the film or IC cavities, each phantom was oriented with the cavity or insert orthogonal to the print bed. The total printing time per phantom was around 30 h depending on the orientation.

**Fig. 2:**
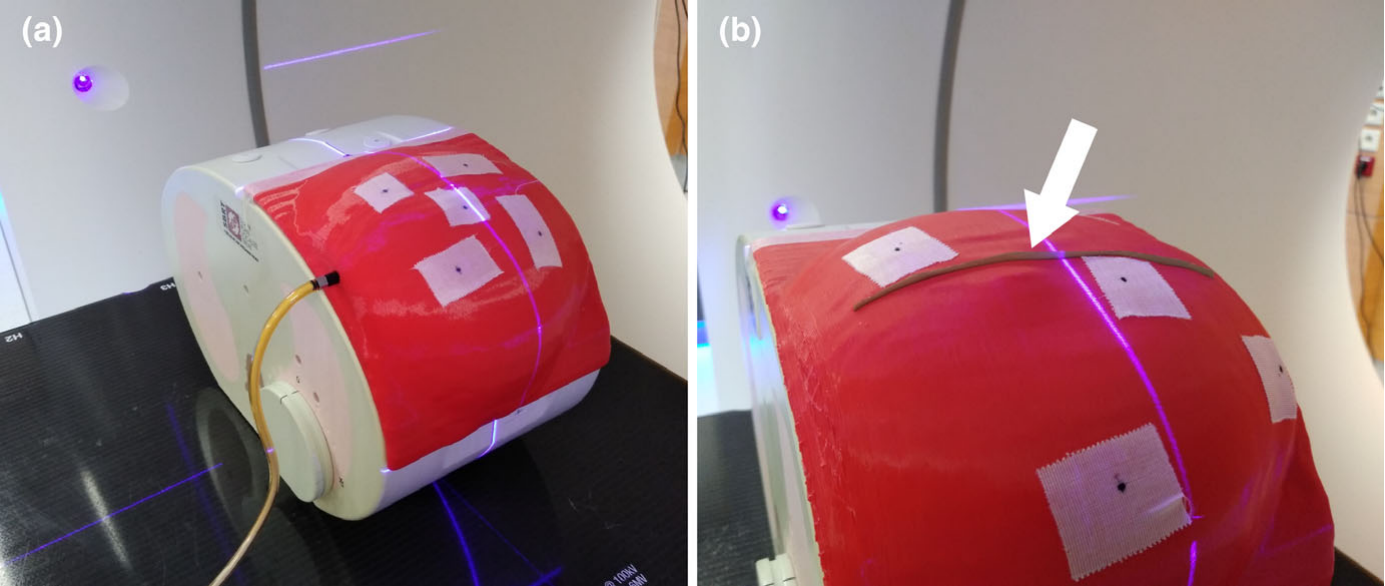
Photographs of the three‐dimensional‐printed breast phantoms attached to the CIRS E2E Thorax SBRT phantom. (a) Ionization chamber phantom with A1SL (Sun Nuclear) inserted. (b) Sagittal film phantom with film inserted. For clarity in a printed version, a white arrow points to the film. Note: see the discussion for the tape with marker points.

The breast phantoms, named E2E breast from hereon, were attached to the E2E SBRT phantom with adhesive tape, Fig. 2. The indentations on the side of the SBRT phantom allow for a unique fit with the 3D‐print. A CT scan was acquired of each phantom at 120 kVp, slice thickness 1 mm on a Somatom Sensation Open. Prior to the delivery of modulated plans, an open field (10 × 10 cm^2^) was delivered on the E2E phantom with an ionization chamber inserted (A1SL, Sun Nuclear with a SNC PC electrometer) to verify the correct CT density modeling of the 80% PLA/air mixture infill. For a dose of 2.66 Gy, the measured and treatment planning system (TPS) calculated values agreed to within 0.4%, therefore not requiring a density override in the TPS.

### 2.B VMAT‐SIB treatment planning and pretreatment plan validation

For verification of the E2E breasts, our VMAT‐SIB treatment protocol was selected. A dose of 2.66 Gy/fraction was prescribed on the boost volume and 2.17 Gy/fraction to the breast for a total of 21 fractions, amounting to 55.86 and 45.57 Gy, respectively. The VMAT‐SIB plans consisted of two partial arcs (from ~300° to ~170° gantry angle) with orthogonal collimator rotations (~10°/280°), based on the work of Virén et al.,^12^ Tyran et al.,^13^ and Nicolini et al.^14^ Plans were created in the Eclipse v15.6 (Varian Medical Systems, Palo Alto, CA, USA) TPS with photon optimizer (PO) v15.6.03 for the Halcyon linac system (Varian Medical Systems). The PO v15.6.03 optimizer allowed for the intensity modulation to be performed independently by both layers of the Halcyon's dual‐layer multileaf collimator system (MLC). Final dose calculation was performed with the AAA v15.6.03 algorithm (dose grid size 1 × 1 × 1 mm^3^).

Six CT scans in treatment position of six random breast cancer patients were used to create VMAT‐SIB plans. Breast volumes ranged from 385 to 1380 cc. To verify the plan delivery of the individual VMAT‐SIB plans, pretreatment verification measurements were performed using portal image dosimetry, ArcCHECK diode array (Sun Nuclear, Melbourne, FL, USA), and both A1SL ionization chamber point dose measurements (Sun Nuclear) and radiochromic EBT3 film measurements (GafChromic, Ashland Specialty Ingredients, Wayne, NJ, USA) in a MultiCube phantom (IBA, Louvain‐La‐Neuve, Belgium).

All films were scanned with a Epson 10000XL flatbed scanner using Epson software without any corrections at a resolution of 150 dpi with 48‐bit color depth, minimally 48 h postirradiation. Film dose calibration was performed using the calibration method of Crijns et al.,^15^ which uses two page‐sized calibration films to simultaneously estimate the dose response of the film and the lateral scan effect. The calibration films were calibrated on a different day than the experimental measurements. Prior to scanning the experimental films, both calibration films were scanned to generate a calibration curve of the day that was used for later dose conversion. Dose conversion was performed using a triple‐channel dose conversion algorithm with lateral scan correction^15^, ^16^ implemented in MeVisLab v2.5 (MeVis Medical Solutions AG, Bremen, Germany). This triple‐channel method combines the scanned optical density in the three color channels (red‐green‐blue) compared to a single channel (usually the red channel) to determine the dose. Using this method dose‐independent effects on film coloring, such as scratches or an uneven film thickness, can be mitigated. An in‐house developed Matlab 2018a (The MathWorks Inc., Natick, Mass., USA) script^17^ was used for comparing the measured dose with the TPS predicted dose, which was exported at a resolution of 0.4 × 0.4 mm^2^. A global rescaling of the film to the TPS predicted dose and a low‐dose exclusion threshold of 10% using a 3% (local)/2 mm γ‐criterion, as per TG‐218 recommendations,^18^ were used. All plans were delivered on a Halcyon linac.

### 2.C Phantom validation with hybrid plan deliveries and an end‐to‐end test

#### 2.C.1 Hybrid plan verification on the E2E breast phantoms

The six patient plans were recalculated on the planning CT of the E2E breasts, as a hybrid plan verification. Online setup was performed with AlignRT (VisionRT Ltd., UK) and verified with kV‐CBCT, as per our image‐guided RT (IGRT) protocol. All plans were verified using both A1SL point dose measurements and radiochromic film measurements. Radiochromic EBT3 films were cut with a Trotec laser cutter (Trotec Laser GmbH, Austria) using a template to match the inserts in the E2E breasts. All pieces were reassembled for scanning, as shown in Fig. S1 of the additional material.

#### 2.C.2 End‐to‐end test with a plan created on the E2E breast phantoms

**Fig. 3:**
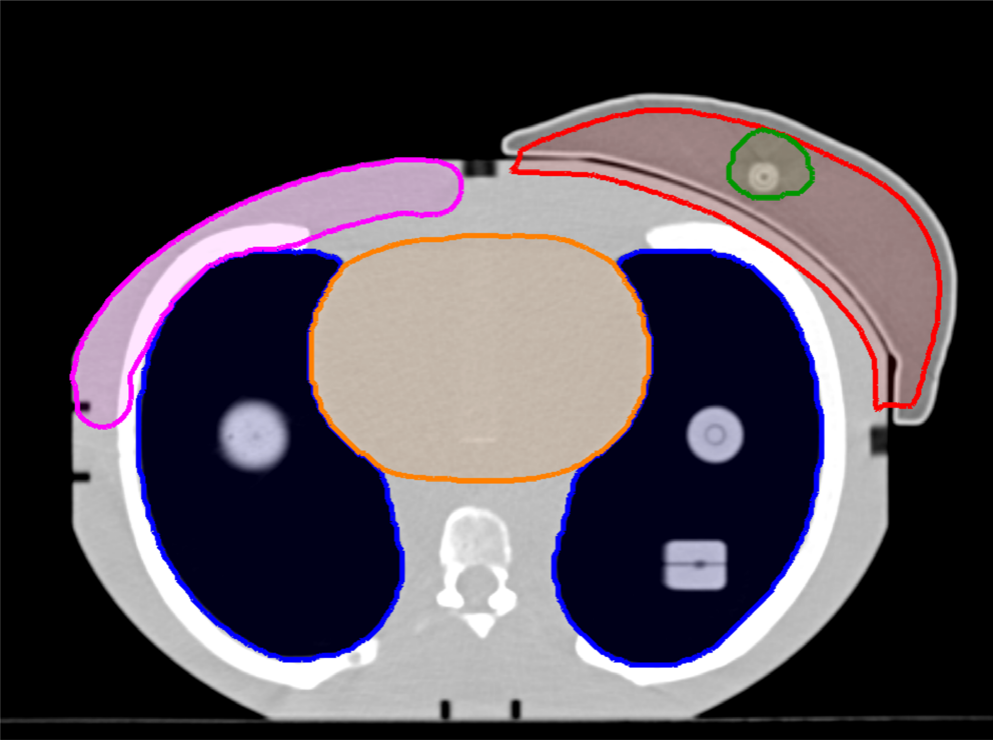
Delineated CIRS phantom with ionization chamber insert three‐dimensional‐printed phantom. Blue: lungs, orange: heart, purple: contralateral breast/chest wall, red: CTV_45.57Gy_, green: CTV_55.86Gy_. The tumor bed was artificially drawn around the active volume of the ionization chamber.

An end‐to‐end test was performed by creating a seventh plan on the E2E breast. The planning CT with the IC E2E breast was delineated according to our departmental protocol, provided organs were present in the CIRS phantom. The following organs at risk (OAR) were delineated: contralateral breast/chest wall, heart, and lungs. The CTV_45.57Gy_ was the entire breast, cropped 5 mm below the skin. A boost volume CTV_55.86Gy_ was artificially drawn around the active volume of the IC, located centrally in the breast, as shown in Fig. 3. PTVs were constructed by extending the CTVs by an isotropic 5 mm margin. A VMAT‐SIB plan was generated using the strategy outlined in section B. Setup was again performed using AlignRT and verified by kV‐CBCT, prior to treatment delivery. Ionization chamber and film measurements were performed.

## 3 RESULTS

### 3.A Pretreatment plan verification

Plan deliverability of the six random patient plans was verified prior to delivery on the 3D‐printed breast phantoms. Point dose differences in the MultiCube were median 0.3% (range −0.5%; 2.4%). Planar γ‐index analysis (3%/2 mm) using portal imaging had a median γ‐index agreement score (γAS) of 100% (range 98.2%–100%). The γAS for ArcCheck was median 92.0% (range 87.3%–95.5%). Multicube film γ‐analysis had median γAS of 93.3% (range 90.6%–98.0%). Point dose, radiochromic film measurements, and portal dosimetry results were within the action limits of the AAPM TG‐218 report (point dose difference < 3% and γAS > 90%) for all the plans and were thus deemed deliverable. One plan fell below the action limits using ArcCheck, failures were detected in the low dose regions (<20%) which was judged clinically acceptable.

### 3.B Hybrid plan verification and end‐to‐end test on the E2E breast phantoms

Results of planar γ‐analysis and point measurements in the E2E breasts are shown in Table 1. Median γAS was 96.4% (range 80.1%–99.7%) for all patients and film orientations, and median relative point dose difference was −1.4% from the predicted dose (range −2.6%; 0.3%).

**Table 1:** Dosimetric film and ionization chamber point measurements for the hybrid plans and end‐to‐end test.

Case	Film			IC point dose
	Coronal	Sagittal	Axial	Diff (%)
	γAS (%)	γAS (%)	γAS (%)	Total
Plan 1	90.3	98.5	91.3	−2.6
Plan 2	96.8	98.5	94	−1.8
Plan 3	98.5	99.7	90.3	−1.4
Plan 4	98.0	99.7	92.3	−1.1
Plan 5	94.4	92.2	97.5	0.3
Plan 6	86.2	97.1	82.7	0.0
E2E breast	80.1	99.7	96.4	−2.3
Median	94.4	98.5	92.3	−1.4

**Fig. 4:**
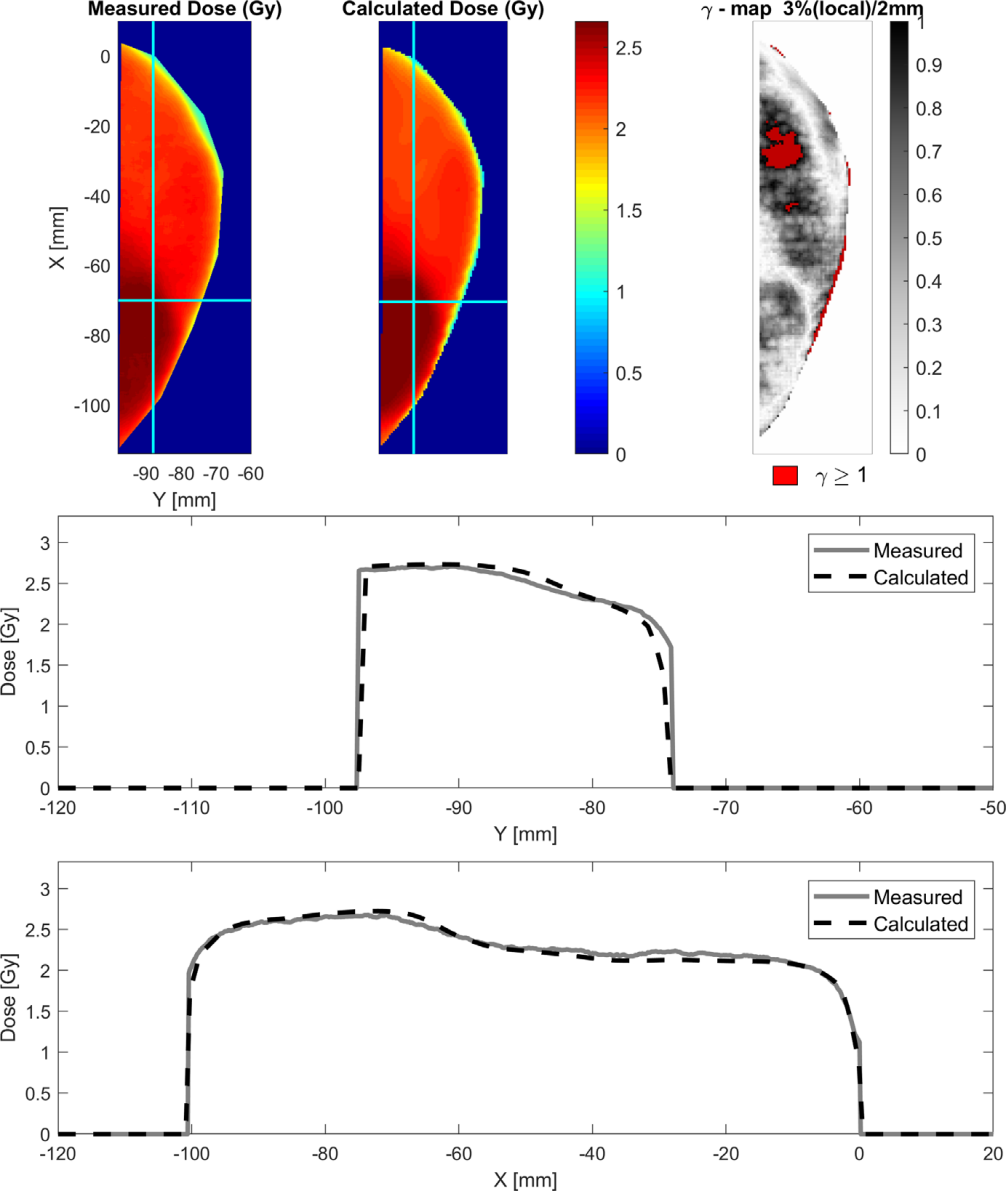
γ‐analysis of the axial film of the VMAT‐SIB plan on the E2E breast. Two profiles (cyan) (inline and crossline) are shown with calculated and measured dose. A γ‐value of 96.4% (with 3% (local)/2 mm and cutoff threshold of 10%) was found.

The γ‐analysis for the axial film of the end‐to‐end plan is shown in Fig. 4. The analysis for a hybrid plan verification on a coronal film is supplied in the additional materials.

## 4 DISCUSSION

In this work we have demonstrated the development of a series of 3D‐printed left breast phantoms which can be attached to an existing commercial dosimetry phantom. These phantoms were validated with hybrid plan measurements and an end‐to‐end test of a VMAT‐SIB treatment technique. The phantoms were setup with surface scanning technology, simulating the clinical practice. The use of the phantoms extends beyond end‐to‐end validation of a breast protocol, as the phantoms are also used as a multipurpose QA tool in our department.

For instance, if routine pretreatment portal dosimetry QA fails, the causes are investigated by delivering the plans on ArcCheck and film inserted into our selection of anthropomorphic phantoms, to which the E2E breasts have been added. Additionally, the phantoms are used during updates of our treatment techniques, ie, a bilateral VMAT treatment technique and VMAT for whole breast and nodal regions.

Not only dose verification is performed with the E2E breasts but they have also functioned as a patient surrogate during the commissioning of a new laser system for virtual simulation of electron boosts. The Horus 5 system (A2J Healthcare) uses five mobile room lasers, which we used to indicate the isocenter and the four corners of the electron field, as can be seen on Fig. 2. The communication of the treatment planning system (Eclipse, Varian Medical Systems) to the laser software and the upgraded dose calculation algorithm (eMC 15.1, Varian Medical Systems) were verified in an end‐to‐end test using both the ionization chamber and film insert phantoms.

The phantoms were fully compliant with the AlignRT surface scanning system, which is increasingly used for setup of breast cancer patients at radiotherapy departments.^5^, ^19^, ^20^ The shape and red color of the PLA resulted in a stable surface registration result. During RTT training and hardware and software upgrade verification of the surface scanning system, we now use the E2E breasts compared to the vender‐provided geometric phantom.

As a validation we chose VMAT‐SIB plans due to the complex dose distribution with steep gradients, however, tangential field‐in‐field or IMRT plans could also be used. We suspect a change in the planning protocol would have no effect on the acceptance of the phantoms in the clinic.

Previous 3D‐printing dosimetry studies focused on developing a complete phantom, usually mimicking head and neck anatomy.^8^, ^21^, ^22^ The authors were only able to find one other paper by Craft et al.^23^ who created a phantom specifically for (postmastectomy) chest wall radiotherapy. In contrast, we aimed to only create an attachment to our in‐house end‐to‐end phantom.

Point doses differ from the calculated value to within 2.6% and film γ‐analysis shows agreement scores above 80.1% for a VMAT‐SIB protocol. We note generally lower agreement scores for the axial film inserts, compared to the sagittal and coronal insert. We suspect this effect to be due to a small air gap (1 mm) around the film due to the slightly larger insert width caused by local warping of the 3D print. In the future, the axial film phantom could be reprinted with care to eliminate warping. The low agreement score for the coronal film of the E2E breast, we suspect to be, due to a slight residual roll rotation of the phantom after setup, as the plan verification gamma agreement scores, using the MultiCube, EPID, and ArcCheck measurements, were above 88%.

We have limited the breast phantoms to a single size. It would, however, be useful to also create a supplementary set of phantoms for very large breasts (e.g., >1800 cc) or pendulous breast to verify the applicability of the VMAT‐SIB technique for more extreme anatomies.

The proposed attachable left breast phantoms allow for a realistic (hybrid) end‐to‐end test of breast radiotherapy protocols. The procedure described in this report can be reproduced by others to create their own breast phantoms, matching in‐house dosimetry equipment.

## CONFLICT OF INTEREST

This work was supported by Varian Medical Systems.

## Supporting information


**Figure S1**. Assembled scanned film.
