# Supplementary material for: Technical Note: Development of 3D‐printed breast phantoms for end‐to‐end testing of whole breast volumetric arc radiotherapy
Source: J Appl Clin Med Phys. 2020 Aug 15;21(8):315–20. doi: 10.1002/acm2.12976 (PMC7484846; doi:10.1002/acm2.12976)
Supplement: Supplementary file 1 — Figure S1. Assembled scanned film. [file ACM2-21-315-s001.docx]

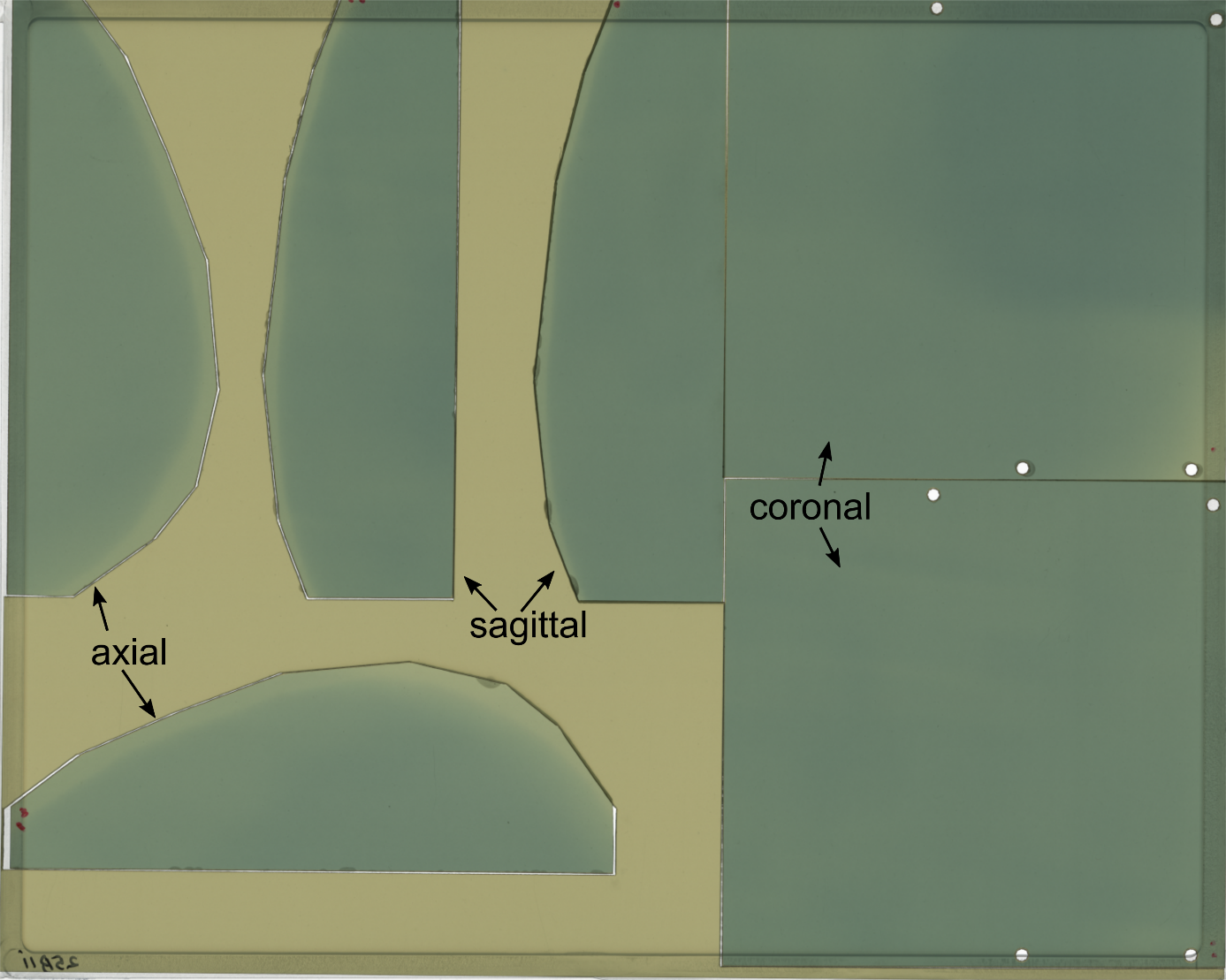


**Fig. 1**. Assembled scanned film. EBT3 films were cut with a Trotec laser-cutter using a template to match the inserts in the breast phantoms. From every film 2 complete sets of inserts (coronal, axial and sagittal) were available. Films were reassembled for every scan.


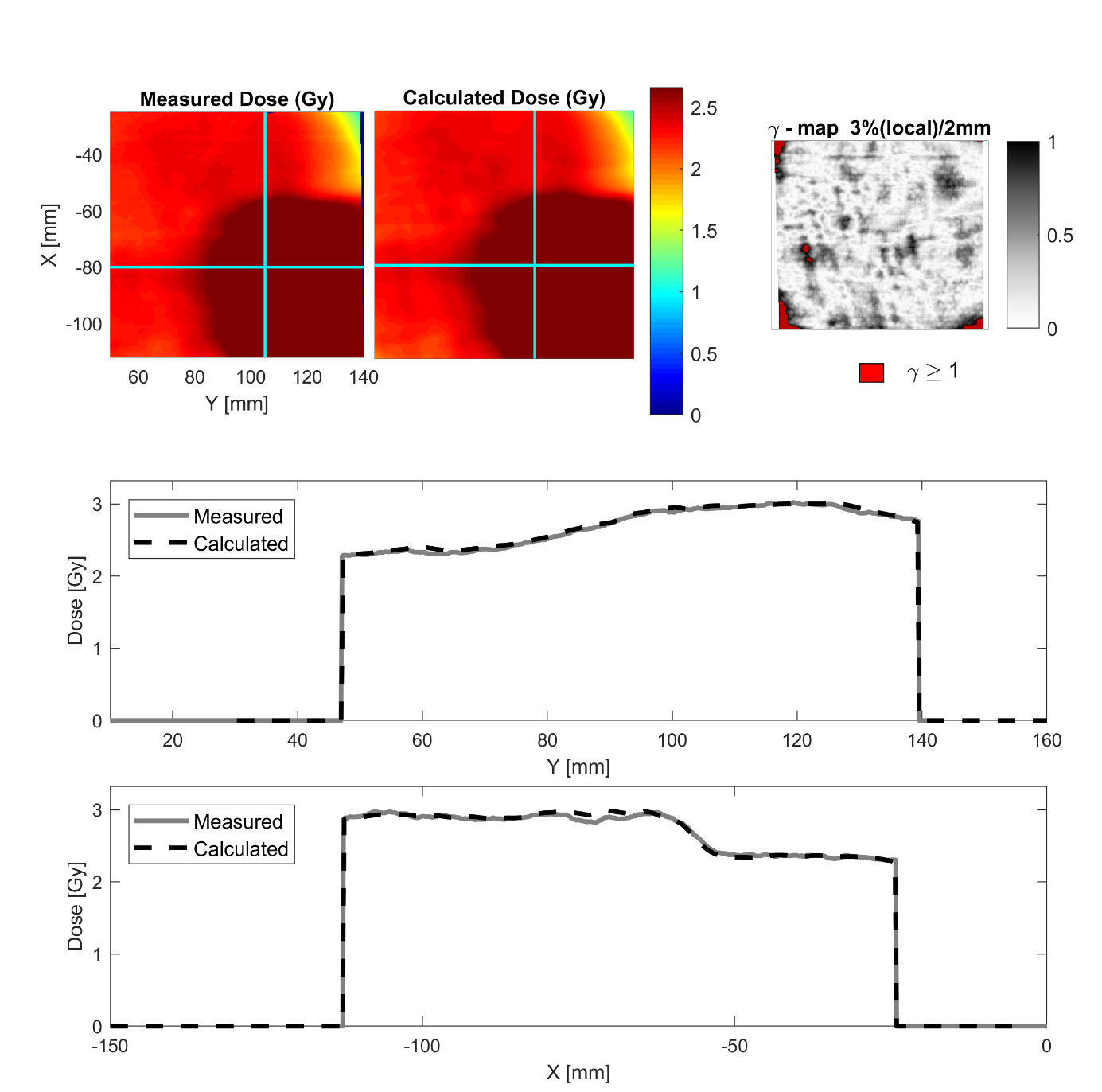


Fig 2. Gamma map and line profiles for the coronal film op a patient plan delivered on the breast phantom.
